# 不同机制所致的血管紊乱化生长的共同干预——安罗替尼治疗右肺鳞癌合并血栓闭塞性脉管炎1例

**DOI:** 10.3779/j.issn.1009-3419.2020.01.10

**Published:** 2020-01-20

**Authors:** 慧娟 郝, 旻 张, 国清 张, 慧 朱, 婺平 包

**Affiliations:** 200080 上海，上海交通大学附属第一人民医院，呼吸与危重症医学科 Department of Respiratory and Critical Care Medicine, Shanghai General Hospital Affiliated to Shanghai Jiao Tong University School of Medicine, Shanghai 200080, China

**Keywords:** 安罗替尼, 血管生长因子, 血栓闭塞性脉管炎, Anlotinib, VEGFR, Thromboangiitis obliterans

## Abstract

**背景与目的:**

肺目前为止血栓闭塞性脉管炎（thromboangiitis obliterans，TAO，又称Buerger disease）仍无理想的治疗方法。安罗替尼作为三线药物，指南推荐用于二线治疗后复发或进展的晚期非小细胞肺癌（non-small cell lung cancer, NSCLC）患者。分析1例右肺鳞癌合并血栓闭塞性脉管炎的治疗困境及疗效评价，为两种疾病的治疗提供新的思路。

**方法:**

回顾性分析了上海交通大学附属第一人民医院呼吸与危重症医学科2018年8月收治的1例右肺鳞癌合并血栓闭塞性脉管炎的诊断、治疗过程，并文献复习。

**结果:**

患者男性，73岁，因“咳嗽、咳痰5月”入院。查体双下肢色素沉着伴足背动脉搏动减弱。既往有吸烟史，可疑血管性间歇性跛行并游走性静脉炎一年余。胸部计算机断层扫描（computed tomography, CT）提示右上肺肿瘤伴阻塞性肺炎，气管镜活检证实为鳞癌；B超提示双上肢及双下肢多发动脉闭塞，下肢深静脉血栓。诊断考虑：右肺鳞癌（T4N2M0，Ⅲb期），体力状况（performance status, PS）评分2分；肺部感染；血栓闭塞性脉管炎。患者外周穿刺置入中心静脉导管（peripherally inserted central catheter, PICC）和静脉输液岗植入术均失败，无法进行化疗。结合文献检索，考虑TAO也可能存在血管内皮细胞生长因子（vascular endothelial growth factor, VEGF）信号通路紊乱，选择安罗替尼（12 mg *qd* *po*）治疗。患者耐受性良好，随访14个月，肺部病变持续空洞化并缩小，同时血栓闭塞性脉管炎好转。

**结论:**

该患者罹患鳞癌合并TAO，安罗替尼治疗可有效控制肺癌增长，同时改善TAO相关症状。安罗替尼可能通过阻断VEGF信号通路，纠正紊乱化生长的血管，而不仅仅是抑制血管生成。

## Abstract (en)

**Background and objective:**

To date, there is no effective treatment for thromboangiitis obliterans (TAO). Anlotinib, as a third-line therapy, is recommended for patients with refractory advanced non-small cell lung cancer (NSCLC). We presented a case report of a patient suffering from right lung squamous cell carcinoma combined with thromboangiitis obliterans, and analyzed the treatment dilemma, which provided a new idea for the treatment of these two diseases.

**Methods:**

A patient of right lung squamous cell carcinoma complicated with TAO was admitted to the department of respiratory and critical care medicine of the Shanghai General Hospital in August 2018. The diagnosis and treatment was retrospectively analyzed, and the literature was reviewed.

**Results:**

The 73-year-old male patient complained of cough and sputum for 5 months and was diagnosed with NSCLC (T4N2M0, stage Ⅲb, performance status score 2) in right upper lung by tracheoscopy biopsy. Pigmentation in both lower extremities accompanied by weakened pulse of dorsal foot artery was confirmed. He had a history of smoking, and suspected vascular intermittent claudication and wandering phlebitis for more than one year. Ultrasound indicated multiple arterial occlusion in both upper and lower extremities and deep venous thrombosis in lower extremities. TAO was diagnosed. Peripherally inserted central catheter (PICC) implantation and intravenous infusion post implantation failed and he could not receive chemotherapy. Vascular endothelial growth factor (VEGF) signal pathway dysfunction is also involved in TAO. Anlotinib (12 mg *qd* *po*) was selected for treatment NSCLC and TAO, accordingly. He had partial response (PR) and the cancer kept stable for 14 months. At the same time, TAO improved.

**Conclusion:**

Anlotinib effectively controlled the growth of NSCLC and improved TAO related symptoms. Anlotinib maybe normalize disordered growth of blood vessels through the VEGF signaling pathway, rather than simply inhibiting angiogenesis.

## 1 病例资料

患者男性，73岁。因“咳嗽、咳痰5月、确诊肺癌1月”于2018年8月9日入院。2018年3月起患者出现反复咳嗽，干咳为主，伴少量黄痰，无明显时相性特点，无胸痛咯血，无呼吸困难，予抗生素（具体不详）治疗未见明显改善。2018年7月24日起出现发热，体温39.5 ℃，外院胸部计算机断层扫描（computed tomography, CT）提示右上肺肿瘤（长径84.8 mm）伴阻塞性肺炎，纵隔肺门淋巴结增大（图 1A）。8月2日-3日行肺穿刺及气管镜，活检结果提示：异型细胞，倾向鳞癌；血管内皮细胞生长因子（vascular endothelial growth factor, VEGF）（-）、ROS1（C-ros oncogene 1 receptor tyrosine kinase）（-）、间变性淋巴瘤激酶（anaplastic lymphoma kinase, ALK）（-）。头部磁共振成像（magnetic resonance imaging, MRI）（增强）及骨正电子发射型计算机断层显像（positron emission computed tomography, PET）-CT排除脑转移和骨转移。为求进一步诊治收入上海市第一人民医院呼吸与危重症医学科治疗。患者既往痛风史6年；曾有吸烟史5年，戒烟14年。否认药物及食物过敏史。入院查体：右上肺呼吸音减低，未闻及明显干湿啰音；双下肢明显色素沉着（图 2）。初步诊断为：（1）右肺鳞癌，T4N2M0，Ⅲb期，表皮生长因子受体（epidermal growth factor receptor, EGFR）（-）、ROS1（-）、ALK（-），体力状况（performance status, PS）评分2分；（2）肺部感染。

**1:**
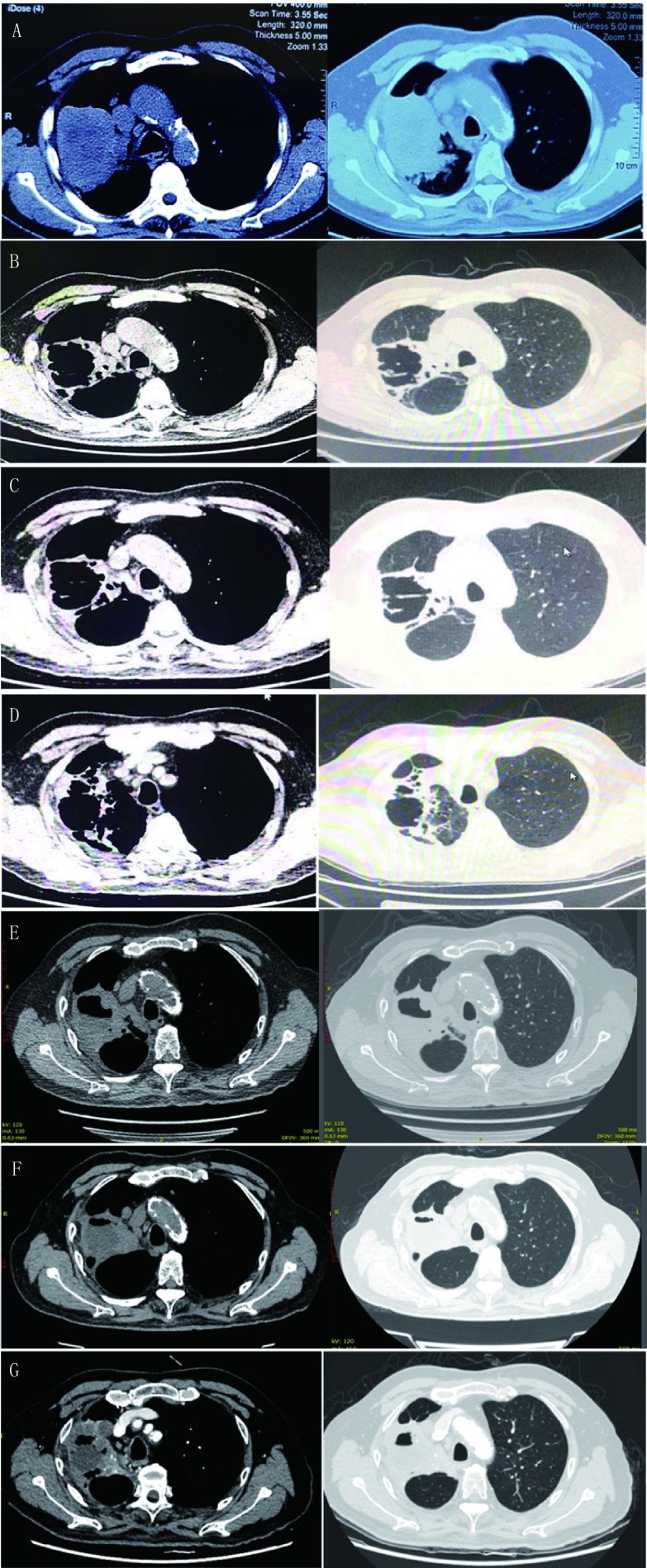
历次胸部CT扫描图像。A：2018年7月25日，右上肺肿瘤（长径84.8 mm）伴阻塞性肺炎，纵隔肺门淋巴结增大；B：2018年9月26日，右上肺肿瘤（长径54 mm）伴空洞形成；C：2018年10月18日，右上肺肿瘤（长径48 mm）；D：2018年12月18日，癌性病灶及空洞维持稳定；E：2019年4月11日，癌性病灶维持稳定空洞缩小；F：2019年7月1日，癌性病灶维持稳定空洞进一步缩小；G：2019年10月12日，癌性病灶及空洞维持稳定。 CT images of the chest. A: right upper lung tumor (major axis 84.8 mm) with obstructive pneumonia and mediastinal lymph node enlargement (Jul 25, 2018); B: right upper lung tumor (major axis 54 mm) with cavitation (Sep 26, 2018); C: right upper lung tumor (major axis 48 mm)(Oct 18, 2018); D: Cancer and cavity remained stable (Dec 18, 2018); E: cancer kept stable and cavity became smaller (Apr 11, 2019); F: Cancer kept stable and cavity shrinks further (Jul 1, 2019); G: cancer and cavity remained stable (Oct 12, 2019). CT: computed tomography.

**2:**
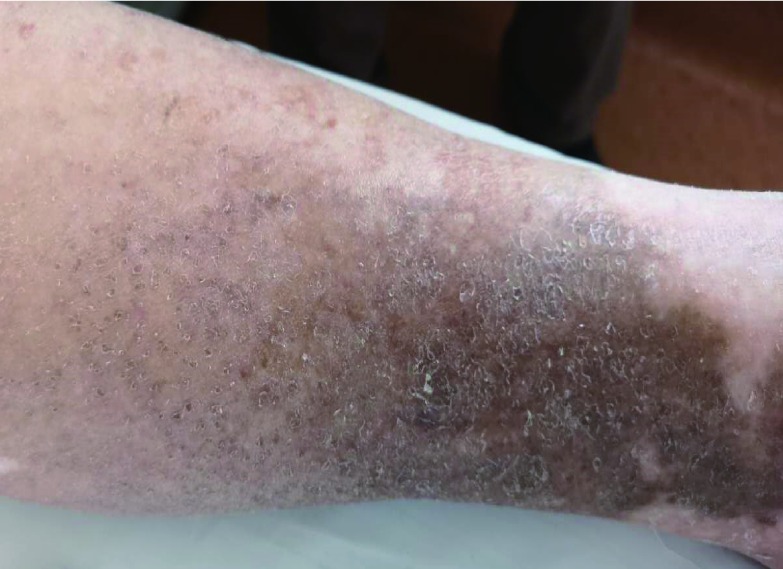
患者双下肢色素沉着明显 pigmentation in both lower limbs

入院后完善心电图、心超、腹部超声、浅表淋巴结超声、肝肾功能、凝血常规均未见明显异常。血常规提示轻度缺铁性贫血。抗核抗体、抗中性粒细胞抗体、双链DNA、类风湿因子、抗链球菌溶血素“O”试验结果阴性。肿瘤指标未见明显异常。予以莫西沙星0.4 g *qd*静脉滴注抗感染治疗，同时先后行中心静脉置管（peripherally inserted central catheter, PICC）、静脉输液港植入术，均插管困难，导致置管失败。追问病史，有可疑血管性间歇性跛行伴游走性表浅静脉炎病史一年余。补充查体发现双下肢足背动脉搏动减弱。2018年8月14日B超提示：两侧下肢深静脉血栓形成；两侧下肢动脉硬化伴斑块形成、右侧股浅动脉及腘动脉闭塞。右侧腋动脉局部闭塞伴周围侧支循环形成可能。

结合病史，患者男性，存在血管性间歇性跛行伴游走性表浅静脉炎病史，吸烟史，查体发现双下肢足背动脉搏动减弱伴色素沉着。超声提示中小动静脉多发性闭塞及栓塞，进一步诊断为：（1）右肺鳞癌（T4N2M0，Ⅲb期），PS评分2分；（2）肺部感染；（3）血栓闭塞性脉管炎。国内外尚无肺癌合并TAO的报道，患者置管失败，肺部病变较大，放疗可能出现严重的局部损伤及放射性肺炎，且放疗可能存在TAO加重的风险，因此不能进行同步放化疗的标准治疗，充分知情并告知大咯血及高血压风险较普通患者更高，患者及家属同意并签名后，予以安罗替尼12 mg *qd*（服2周，停1周）口服治疗，考虑抗凝治疗可能增加大咯血风险，未抗凝。治疗1周后，患者有血压升高（140-155 mmHg/80-95 mmHg），予以奥美沙坦和硝苯地平控释片治疗后维持稳定；用药2周后，患者出现右下肢足跟部皮肤破溃出血，无疼痛，查体足背动脉搏动正常，无游走性表浅静脉炎，无肢端坏死，同时间歇性跛行改善。考虑出血可能与血管再通有关，故未停用安罗替尼，给与局部纳米银材料换药覆盖。2018年9月26日复查胸部CT提示右上肺肿瘤（长径54 mm）伴空洞形成（图 1B）。2018年10月18日复查胸部CT提示右上肺肿瘤（长径48 mm）伴空洞形成（图 1C）。足跟部皮肤破溃改善，且复查B超提示双下肢动脉较前无明显变化，双下肢深静脉血栓消失。2018年12月18日（图 1D）、2019年4月11日（图 1E）、7月1日（图 1F）及10月12日（图 1G）复查胸部CT提示癌性病灶及空洞维持稳定。截至投稿，仍维持安罗替尼治疗，并奥美沙坦和硝苯地平控释片控制血压。

## 2 讨论

盐酸安罗替尼是一种新型小分子多靶点酪氨酸激酶抑制剂，能有效抑制血管内皮细胞生长因子受体（vascular endothelial growth factor receptor, VEGFR）、血小板衍生生长因子受体（platelet-derived growth factor, PDGF）、成纤维生长因子受体、干细胞生长因子受体等激酶的活性，进而发挥抗肿瘤血管生成和抑制肿瘤生长的作用^[1, 2]^。盐酸安罗替尼治疗晚期非小细胞肺癌（non-small cell lung cancer, NSCLC）专家共识^[3]^指出，安罗替尼单药适用于既往至少接受过2种系统化疗后复发或进展的局部晚期或转移性NSCLC患者，能显著延长晚期NSCLC患者的总生存期（overall survival, OS）和无进展生存期（progression-free survival, PFS）。

血栓闭塞性脉管炎是一种少见的非动脉粥样硬化性慢性复发性节段性炎症性疾病，主要累及四肢的中、小动脉和静脉，下肢多见。TAO是由于不明原因的小动脉痉挛和血栓形成造成闭塞，致使局部缺血，半数伴有雷诺现象，多见于中低社会经济地位的青壮年吸烟男性。临床表现为患肢缺血、疼痛、间歇性跛行、足背动脉搏动减弱或消失和游走性表浅静脉炎，严重者有肢端溃疡和坏死。血栓闭塞性脉管炎是血管外科难治性疾病之一，其治疗虽然方法多种多样，但目前为止仍未找到一种理想的治疗方法^[4]^，而关键是无法阐明TAO的发病机制。

在成年人中，血管保持静止，生理条件下很少形成新的分支。血管网络是在胚胎发生早期由血管发生（vesculogenesis）和血管生成（angiogenesis）的过程联合形成的。血管在发育、组织修复或疾病状态下的生长过程中涉及内皮细胞的萌发、迁移和增殖，受多种因素调控。血管形成是促血管形成因子和抑制因子协调作用的复杂过程，正常情况下二者处于平衡状态，一旦此平衡打破就会激活血管系统，使血管生成过度或抑制血管系统使血管退化。

VEGF是一种能够控制内皮细胞增殖、迁移、浸润及生存的重要的血管生成因子，常在NSCLC内过度表达，已成为一重要的治疗靶点。VEGF包括VEGF-A、VEGF-B、VEGF-C、VEGF-D和VEGF-E，以及胎盘源性生长因子（placental growth factor, PIGF），VEGF与位于内皮细胞表面的VEGFR结合，进而激活细胞内下游信号通路，刺激内皮细胞增殖分化成熟迁徙，生成新生血管。VEGFR包含3种亚型，分别为VEGFR-1、VEGFR-2和VEGFR-3三种亚型。

为了支持癌细胞的高增殖率，肿瘤需要快速生成新的血管网络。然而，新生的肿瘤血管并不成熟，存在血管功能不全^[5]^。肿瘤血管功能不全影响肿瘤微环境，可导致缺氧、免疫细胞浸润和活性降低、转移性传播风险增加。肿瘤血管发育不正常，部分原因是肿瘤和基质细胞分泌的生长因子水平异常，其中VEGF起着关键作用，其他生长因子包括血管生成素、PDGF、转化生长因子等对肿瘤异形血管网的形成也具有重要影响^[6]^。抗血管生成治疗可以纠正肿瘤血管的结构和功能缺陷，这一过程被称为血管正常化^[7, 8]^。血管正常化的概念表明，化疗和放疗联合VEGF信号抑制剂的疗效可能是对血管的成熟效应。

安罗替尼对VEGFR-2的抑制活性最强，主要结合在VEGFR-2和c-Kit激酶的ATP结合域，对于干细胞因子受体c-Kit也有强抑制活性，既能全面抑制肿瘤血管新生，也能通过c-Kit等激酶干预肿瘤细胞本身多个生物学过程的双重功能。安罗替尼通过抑制VEGF通路，改善VEGF通路的失衡状态，对肿瘤及TAO均能发挥治疗作用。研究^[9]^表明阻断PDGF也可以使异常的肿瘤血管退化，而正常的血管不受影响，从而改善肿瘤血管的密度，增加灌注，改变肿瘤微环境。

Matsui等^[10]^研究发现TAO患者血清中VEGF水平显著高于对照组。随后，Hewing等^[11]^研究表明，与吸烟及非吸烟健康人群相比，TAO患者外周血单个核细胞中CD34^+^VEGFR2^+^祖细胞比例明显升高，且与对照组相比TAO患者的血清中VEGF水平往往更高。而且TAO患者中VEGFR-1较对照组显著升高，可能与血管内层中形成血管滋养管有关，血管滋养管的形成抑制血管舒张。因此，TAO患者可能存在VEGF通路紊乱，使其血管生成受抑，且血管功能障碍。通过纠正VEGF通路导致的血管紊乱，重建并恢复血管正常化，可能是TAO治疗的新靶点。

此例患者老年男性，隐匿起病，慢性病程，右肺鳞癌的临床及病理诊断明确，血栓闭塞性脉管炎诊断明确。安罗替尼（12 mg *qd* *po*）治疗，随访14个月，患者耐受良好，近一年维持稳定。病例证实安罗替尼对于鳞癌有积极的治疗作用，同时没有加重血栓闭塞性脉管炎，甚至使其出现一定程度的改善，提示安罗替尼对于肿瘤性血管生成和闭塞性脉管炎中血管紊乱均有调节作用，其作用机制很可能是使血管生成正常化，而不仅仅是阻断血管生成。

综上所述，此案例报道的意义在于，首先，鳞癌合并TAO的患者接受安罗替尼治疗后应答良好，并未出现不可控的大出血和血压升高，为合并这一疾病的鳞癌患者的临床治疗提供了信心。其次，更值得关注的是，此案例为TAO患者存在促血管形成因子和抑制因子失衡提供证据。最后，安罗替尼目前主要作为抑制血管生成药物用于临床抗肿瘤治疗，其抗血管生成作用使血管正常化的潜在作用机制，可能有助于TAO患者的VEGF通路异常及血管紊乱化生长的逆转。具体的机制及作用疗效有待进一步基础及临床研究的验证。
